# Multidirectional traction-assisted rectal endoscopic submucosal dissection with prophylactic Glubran 2 nebulization in a high-risk bleeding patient receiving ongoing anticoagulant therapy

**DOI:** 10.1055/a-2953-7101

**Published:** 2026-09-17

**Authors:** Giuliano Francesco Bonura, Giulia Bertoncini, Federica Indulti, Giovanni Aragona, Chiara Salvatore, Paola Soriani, Mauro Manno

**Affiliations:** 1 Gastroenterology and Digestive Endoscopy Unit 18067 Azienda USL ModenaModenaItaly; 2 Gastroenterology and Hepatology Unit 154818 Azienda USL PiacenzaPiacenzaItaly

Endoscopic submucosal dissection (ESD) is the standard technique for en-bloc resection of large colorectal lesions. 1​ However, ESD may be technically challenging in the presence of submucosal fibrosis and poor visualization of the dissection plane. 2​ Traction-assisted methods improve exposure of the submucosal layer and facilitate safer dissection. 3​ Delayed bleeding remains one of the most relevant adverse events after rectal ESD, particularly in elderly patients receiving anticoagulant therapy, and no standard prophylactic strategy has yet been established. 4​

We report the case of an 85-year-old man with anemia and hematochezia referred for ESD of a 70-mm JNET2B rectal non-granular pseudo-depressed laterally spreading tumor ( Fig. 1 ). His medical history included atrial fibrillation on direct oral anticoagulant therapy, ischemic heart disease, hypertension, and chronic kidney disease. The procedure was performed under spinal anesthesia. 5​ Because of suspected submucosal fibrosis, traction-assisted ESD was planned ( Video 1 ).

**Fig. 1:**
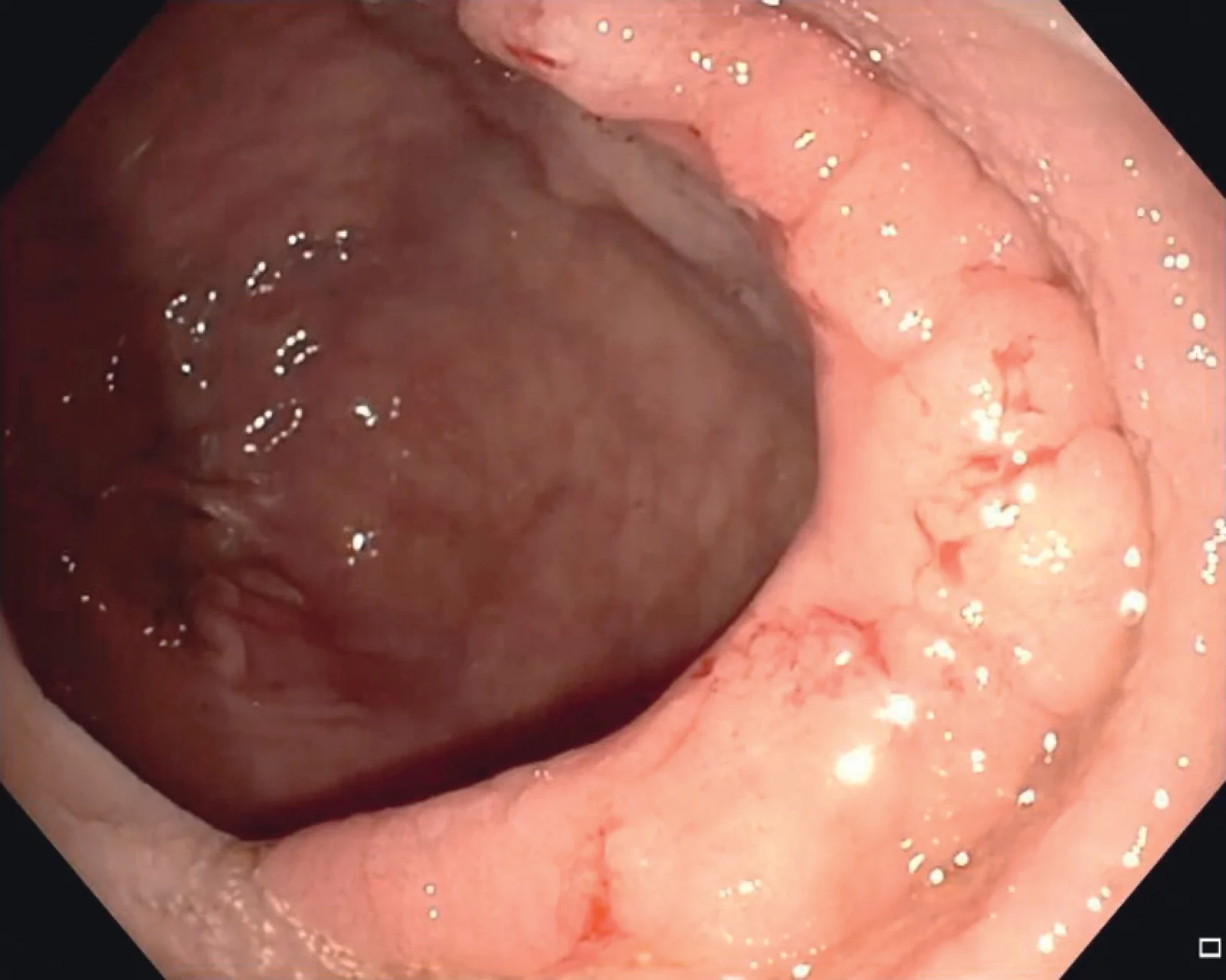
Rectal non-granular pseudo-depressed laterally spreading tumor.

**Video 1** Multidirectional traction-assisted rectal endoscopic submucosal dissection with prophylactic Glubran 2 nebulization.


After circumferential mucosal incision using a HybridKnife flex T-type (ERBE, Tubingen, Germany), the SureTrac traction system (Micro-Tech, Europe) was applied. This device consists of a primary clip connected to an elastic silicone band with four rings, allowing dynamic multidirectional traction and adjustment of the traction angle during dissection ( **Figs.** 2 **and** 3 ). The resulting tissue elevation provided excellent visualization of the submucosal layer, enabling safe en-bloc resection without intraprocedural adverse events ( Fig. 4 ). The total procedure time was 80 minutes.

**Fig. 2:**
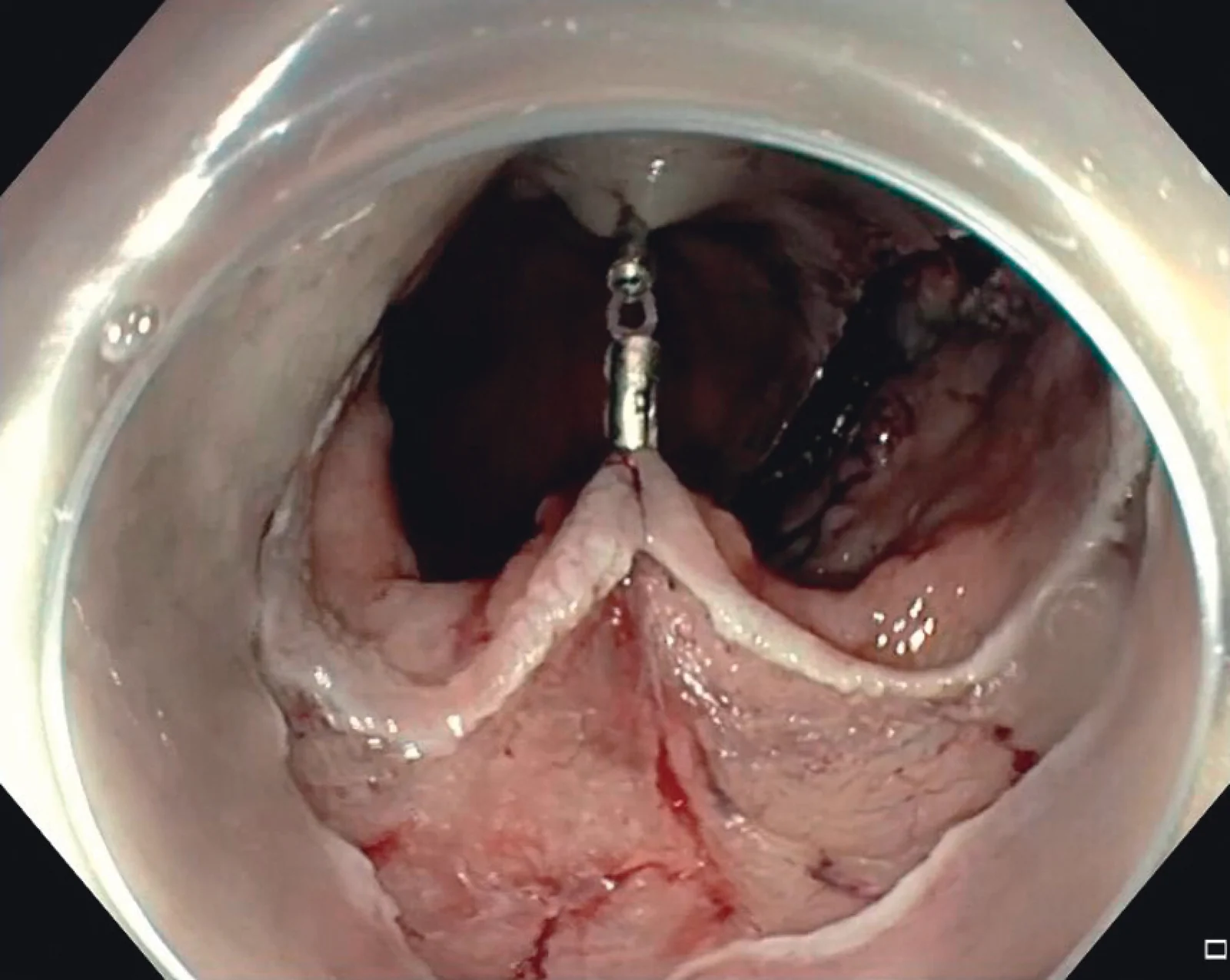
First traction using the Suretrac system.

**Fig. 3:**
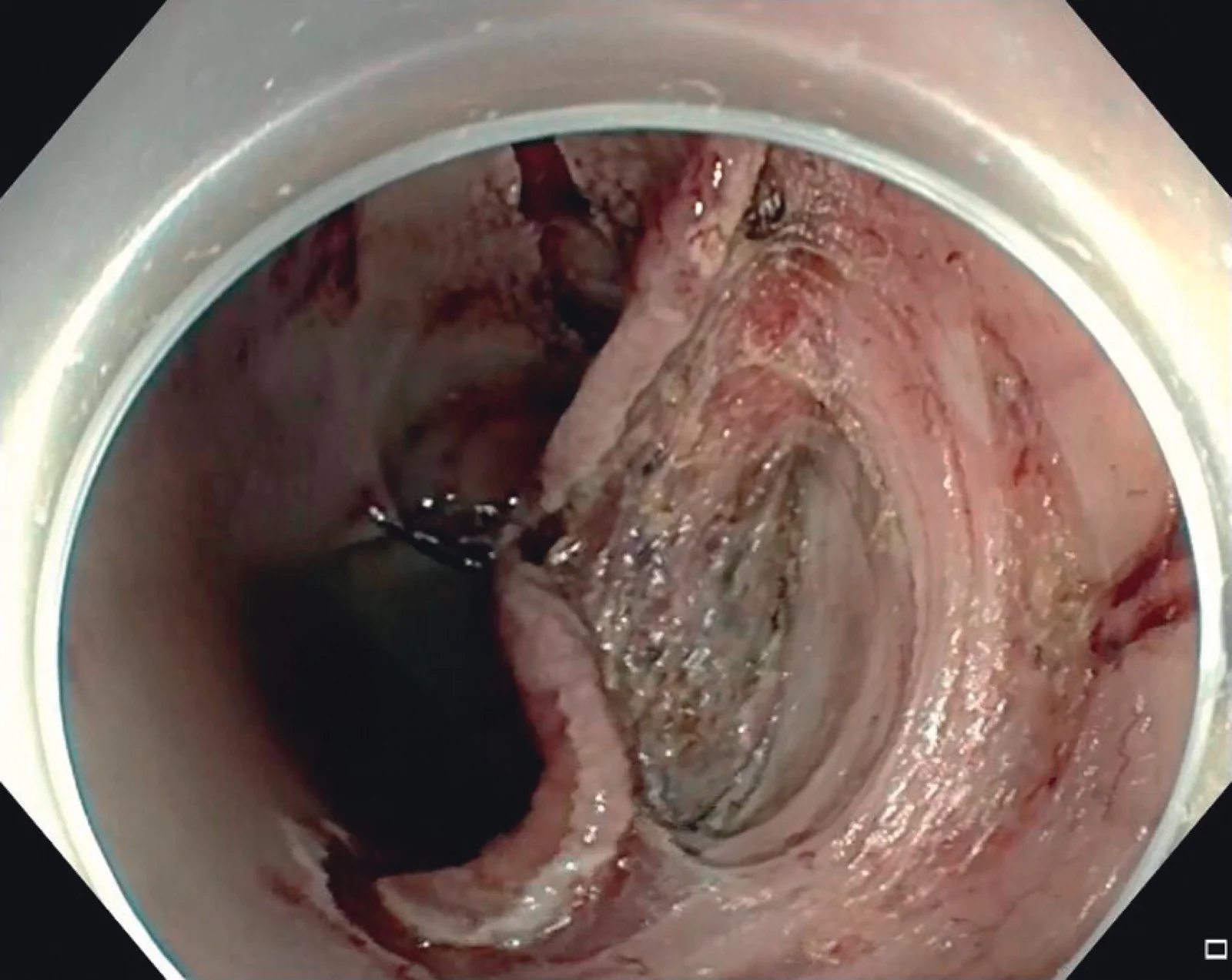
Second traction using the Suretrac system.

**Fig. 4:**
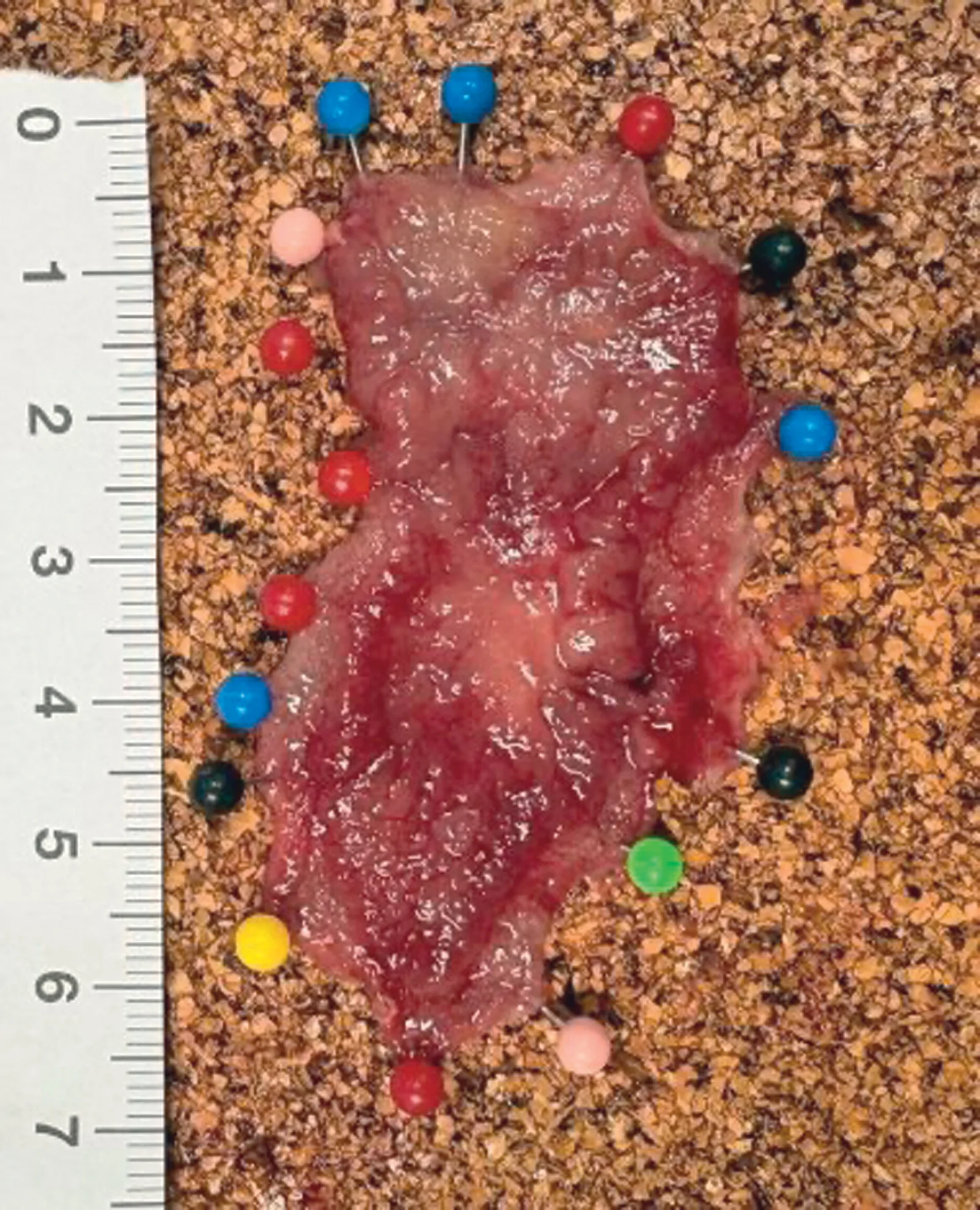
The en bloc resected specimen.

Considering the patient’s high bleeding risk and the need for early anticoagulation resumption, prophylactic Glubran 2 nebulization using an EndoNeb system (GEM, Italy) was applied over the resection bed ( Fig. 5 ). The patient was discharged the following day without complications or delayed bleeding. Histopathology revealed submucosal adenocarcinoma (pT1b, sm2, L0, V0, Bd1, R0, and G1). Multidisciplinary evaluation recommended clinical and endoscopic follow-up only.

**Fig. 5:**
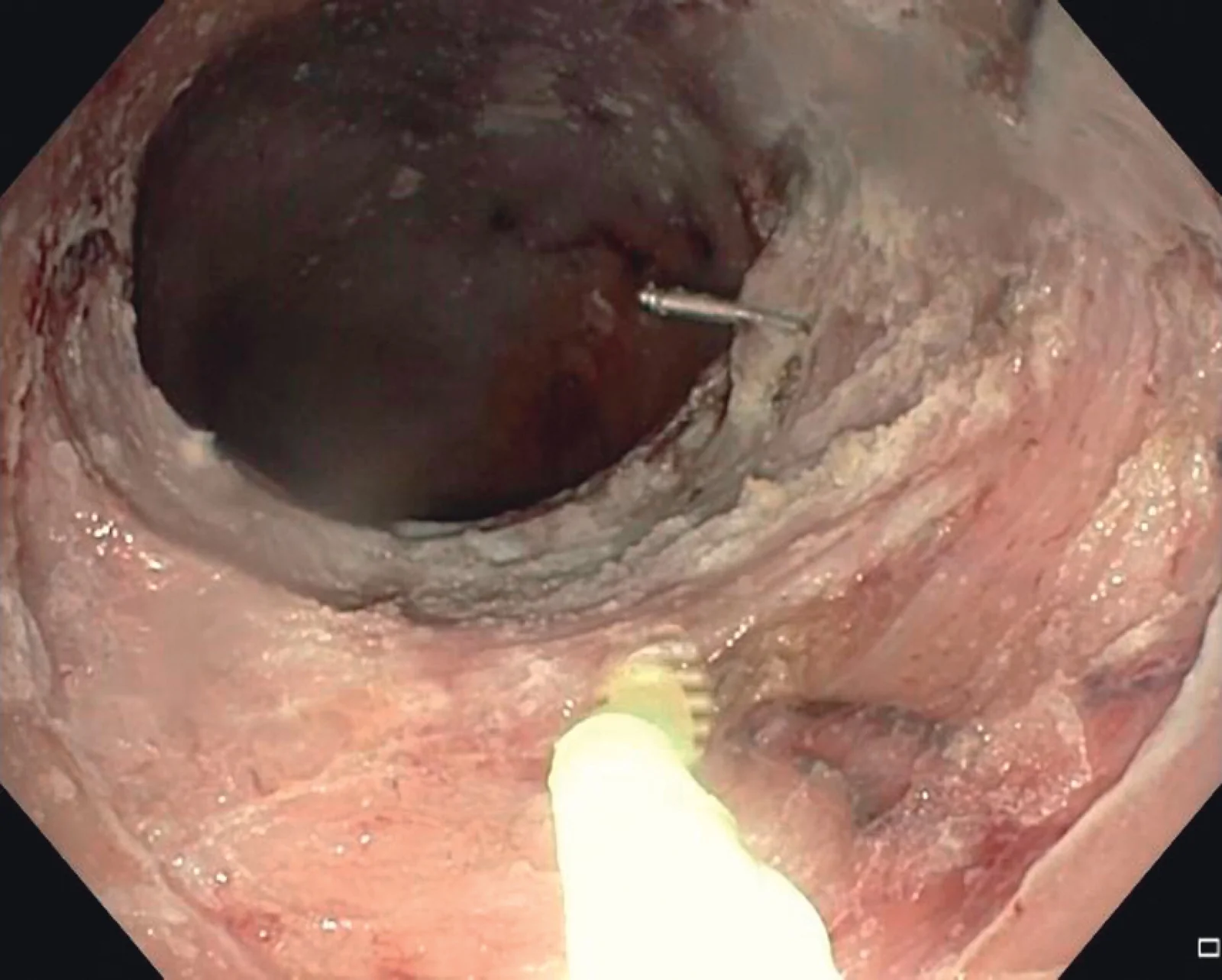
Nebulization of Glubran 2 using an EndoNeb system over the resection bed.

This case highlights the usefulness of multidirectional traction-assisted ESD with SureTrac and suggests prophylactic Glubran 2 nebulization with the EndoNeb system as a promising strategy to reduce delayed bleeding in high-risk patients.

UCTN Code: Endoscopy_UCTN_Code_TTT_1AQ_2AC

## Declaration of GenAI Use

Generative artificial intelligence (ChatGPT) was used solely for English language translation and linguistic assistance during the preparation of this manuscript.
